# Irrelevant sound interference on phonological and tonal working memory in musicians and nonmusicians

**DOI:** 10.1186/s41155-018-0114-z

**Published:** 2019-01-18

**Authors:** Ana Clara Naufel Defilippi, Ricardo Basso Garcia, Cesar Galera

**Affiliations:** Departamento de Psicologia, Faculdade de Filosofia, Ciências e Letras de Ribeirão Preto, Universidade de São Paulo, Av. Bandeirantes 3900, Ribeirão Preto, SP 14040-901 Brazil

**Keywords:** Memory, Short-term memory, Working memory, Tonal memory, Phonological memory, Professional musicians

## Abstract

**Background:**

Working memory refers to the cognitive system responsible for the temporary storage and maintenance of information, but it remains controversial whether overlapping processes underlie the temporary retention of verbal and musical information such as words and tones.

**Methods:**

Participants with little or no musical training (*n* = 22) and professional musicians (*n* = 21) were administered four memory tasks. Two tasks (tone sequence recognition and pseudoword sequence recall) aimed at comparing groups’ performance for tonal or phonological material separately. Other two memory tasks investigated pseudoword and tone recognition under three conditions during the retention interval (silence, irrelevant words, or irrelevant tones).

**Results:**

Musicians were better than nonmusicians in tone sequence recognition but not in pseudoword sequence recall. There were no interference effects of irrelevant tones or words on pseudoword recognition, and only irrelevant tones significantly interfered with tone recognition.

**Conclusions:**

Our results offer further support that tone recognition is specifically impaired by irrelevant tones, but irrelevant words did not disrupt pseudoword or tone recognition. Although these results do not reflect a double-dissociation pattern between phonological and tonal working memory, they provide evidence that temporary retention of tonal information is subject to specific tonal interference, indicating that working memory for tones involves specific processes.

## Background

The concept of working memory (WM) refers to the cognitive system responsible for the temporary storage and processing of information (Baddeley, 2007, 2012). A controversial issue is whether overlapping processes underlie the temporary retention of verbal and musical information such as words and tones (Alley & Greene, 2008; Atherton et al., 2018; Baddeley, 2012; Benassi-Werke et al., 2012; Koelsch et al., 2009; Schendel & Palmer, 2007; Schulze & Koelsch, 2012; Schulze et al., 2011; Williamson et al., 2010a; Williamson et al., 2010b). Although some authors had initially claimed that memory for tones requires specific processes (Berz, 1995; Deutsch, 1970), tonal memory seems to be linked to musical expertise and a clear dissociation between phonological and tonal WM may be not apparent in nonmusicians (Pechmann & Mohr, 1992; Schulze & Koelsch, 2012; Schulze et al. 2011; Williamson et al., 2010a).

The interference paradigm provides a basis for investigating whether overlapping or dissociated processes underlie the temporary retention of phonological and tonal information (Williamson et al., 2010b). That is, the concurrent presentation of specific types of auditory material (such as speech or music) during WM tasks may shed light on the cognitive resources shared by the WM task and the processing of distracting stimuli. In the present study, we investigated recognition memory for phonological and tonal stimuli under different conditions of distracting stimuli (irrelevant words or tones).

Research on this topic typically requires the serial recall of visually presented linguistic items that are studied in a silent condition or with the simultaneous presentation of irrelevant sounds (tones, speech, vocal, or instrumental music) that the participants are instructed to ignore. The findings showed that WM performance is impaired significantly by irrelevant speech, whether from the same or different language as the memorized words (Colle & Welsh, 1976; Salame and Baddeley, 1982), and that interference does not depend on the phonological similarity between memorized and interpolated items (Jones & Macken, 1995; Larsen et al., 2000; LeCompte & Shaibe, 1997; but see Eagan & Chein, 2012). In addition, phonological WM is also impaired by tones, vocal, and instrumental music (Alley & Greene, 2008; Iwanaga & Ito, 2002; Jones & Macken, 1993, 1995; Salamé & Baddeley, 1989).

The source of irrelevant sound interferences (by verbal or musical stimuli) has been considered controversial and may be related to different processes (Baddeley, 2007, 2012; Chein & Fiez, 2010; Eagan & Chein, 2012; Jones & Tremblay, 2000). One possibility may be that irrelevant sounds somehow disrupt phonological codes within WM (interference-by-content), but interferences seem to concern serial order processes (interference-by-process), that is, irrelevant changing sounds are likely to interfere with seriation processes in WM (Beaman & Jones, 1997, 1998; Jones & Macken, 1993; Jones et al., 1992; Jones & Tremblay, 2000; Page & Norris, 2003; Tremblay et al., 2012). In fact, there is scarce evidence that irrelevant speech may be associated with interference-by-content in phonological WM, for example, by disrupting the representation of a single verbal stimulus (e.g., a word or a pseudoword) rather than of a sequence of stimuli. Eagan and Chein (2012) found that a high-overlap of phonetic features between the memorized and interpolated verbal sequence produced the stronger memory disruption in comparison with the low-overlap condition. Atherton et al. (2018) found that the recognition of a single word is disrupted more strongly by interpolated words than by musical chords. The results from both studies support the view that phonological WM may also be prone to trace disruption and interference-by-content. In the present study, we further investigated this issue by using a pseudoword recognition task with interpolated words or tones. That is, by using a phonological WM task without a serial order component, the possibility of interference-by-process was reduced, and we could search for evidence of interference-by-content.

When it comes to the temporary retention of nonlinguistic-auditory information such as tones, there is evidence for a highly specific type of auditory interference without a serial order memory component. Deutsch (1970) used a recognition procedure in which one tone was presented for memorization, followed by a retention interval of 5 s and the presentation of a test tone. The participants were instructed to ignore a sequence of six tones or digits presented during the retention interval. The results showed that only interpolated tones led to a higher rate of errors, while few errors occurred with interpolated digits. A subsequent study by Pechmann and Mohr (1992) employed a similar tone recognition procedure and compared retention intervals filled either with irrelevant tones, speech, or visual material, under both attended and unattended conditions. Interestingly, they also compared musicians and nonmusicians to investigate whether the findings could be generalized to populations differing in musical expertise. For both groups of participants, the irrelevant tones disrupted tone recognition in a similar way, but only for nonmusicians, the interpolated verbal (both attended and unattended conditions) and visual material (only attended condition) had disruptive effects, indicating that tone recognition was disrupted both by attentional and modal interference.

Following the account by Deutsch (1970) and Pechmann and Mohr (1992), one possibility is that disruption of tone recognition results from retroactive interference by interpolated tones on the memorized tone. Another possibility is that disruption results from perceptual processes, such as grouping (temporal proximity) and coherence (spectral similarity), that organize the incoming sounds into perceptual streams, reflecting on the representations within memory and impacting on the retrieval of items within or between streams (Jones et al., 1997). In fact, Jones et al. (1997) found evidence that temporal proximity and spectral similarity influence the level of disruption by interpolated verbal and tonal stimuli (see also Semal et al., 1996); nevertheless, disruption was still significant even after reducing perceptual grouping, suggesting that it may also derive from interference on memory codes, for example, by feature overwriting and updating (Mercer & McKeown, 2010a, 2010b). In sum, evidence indicates that tone recognition is prone to interference-by-content.

More recently, Williamson et al. (2010b) developed a visual-auditory recognition task in which sequences of letters and musical notes were presented visually in a computer display, the participants (amateur musicians) being instructed to encode the visual stimuli into an auditory format in their minds. The retention interval was followed by an auditory sequence (spoken letters or musical tones) for a same or different judgment. The results showed a double-dissociation pattern, that is, irrelevant tones impaired performance for tonal stimuli, whereas irrelevant speech impaired performance for verbal stimuli, suggesting that different processes are involved in the retention of musical and verbal information, at least in the case of musicians. However, it is difficult to generalize such results to individuals without musical knowledge as the experimental procedure involved visual presentation of musical notes and required the knowledge of musical notation. Finally, Atherton et al. (2018) investigated the recognition of a single word or chord in conditions of silence or irrelevant words or chords during the retention interval, and their results showed strong interference effects from matching irrelevant stimuli (i.e., word-words and chord-chords), although mismatching irrelevant stimuli (word-chords and chord-words) produced a smaller significant interference, indicating that the cognitive resources implied in word and chord recognition partially overlap.

In sum, the hypothesis that working memory has dissociated systems for tonal and phonological information deserves additional investigation, for example, by using tonal and phonological recognition tasks without a serial order component, as irrelevant sound interference has been observed in simple recognition tasks in tonal memory, but with multiple stimuli in free or serial recall of word lists. Furthermore, the comparison between musicians and nonmusicians is particularly important to allow a better understanding about the generality of any eventual interference effects in populations differing in musical expertise. This issue is relevant because musical expertise may affect not only the degree of performance in verbal and tonal tasks, but also the degree of susceptibility to interference by irrelevant stimuli. In fact, evidence shows that musical training is associated with better performance in a range of musical and cognitive tasks, including verbal memory and executive functions (Chan et al., 1998; Franklin et al., 2008; George & Coch, 2011; Hansen et al., 2013; Ho et al., 2003; Jakobson et al., 2008; Roden et al., 2014).

In the present study, we investigated whether a double-dissociation pattern would emerge using recognition tasks (without a serial order component) for tone or pseudoword under conditions of silence, speech, or tonal interference. A pseudoword recognition task seemed adequate because pseudowords are word-like verbal stimuli without meaning representation in long-term memory, so that recognition is likely to be phonologically based. In this way, it was possible to test whether irrelevant sounds (specially speech) may disrupt phonological codes within the phonological store. According to the double-dissociation hypothesis of separate phonological and tonal WM components, tone recognition would be impaired by irrelevant tones and not by irrelevant words; conversely, pseudoword recognition would be impaired by irrelevant words and not by tones.

## Method

### Ethics

The present study was conducted in accordance with the national legislation regarding the assessment of human volunteers. Ethical approval for the present study was given by the University of São Paulo FFCLRP Research Ethics Committee (process number 081/2002 – 2002.1.1709.59.7).

### Participants

The sample size was defined prior to data collection to include at least 18 participants per group. A total of 43 individuals participated in this study. Twenty-two nonmusicians (mean age = 25.5 years, SD = 6.9), undergraduate and graduate students reporting little or no experience with a musical instrument, were recruited in the university campus (mean years of musical experience = 1.7, SD = 2.2). Twenty-one musicians (mean age = 29.5 years, SD = 10.6) were recruited in local conservatories and universities: Only professional musicians with a minimum of 5 years uninterrupted of practice with musical instruments were selected (mean years of musical experience = 19.4, SD = 11.1), and these criteria are in line with the specialized literature in music psychology (Zhang, Susino, McPherson, & Schubert, in press). In this group, the musicians’ principal instruments were piano (*n* = 9), guitar or electric guitar (*n* = 6), bass (*n* = 2) accordion (*n* = 1), keyboards (*n* = 1) saxophone (*n* = 1), and violin (*n* = 1). Musicians reporting absolute pitch were not included in the sample.

We ensured that participants met specific criteria to enter the study: they were not on medication, did not report any neurological or psychological problem, and had normal hearing according to the audiological assessment. Pure-tone threshold audiometry was conducted according to the American Speech-Language-Hearing Association (ASHA) guidelines (ASHA, 1978). Air-conduction thresholds were measured for each ear at frequencies of 0.25, 0.5, 1, 2, 3, 4, 6, and 8 kHz. The participants with thresholds up to 25 dB in all the given frequencies were considered without hearing loss (Davis & Silverman, 1978). In addition, speech audiometry was conducted to assess speech recognition threshold (SRT) and speech discrimination score (Pereira & Schochat, 1997; Santos & Russo, 1986). An SRT value up to 10 dB above the participant pure-tone threshold was considered within normality. Speech discrimination was performed 40 dB above the participant pure-tone threshold, and it was expected a score of correct responses equal to or greater than 92%. In total, four individuals who volunteered to participate were not included in this study, two of them for presenting some degree of hearing loss (and they were advised to consult a specialist) and two of them for being amateur rather than professional musicians.

### Materials and procedure

The experimental instructions and all the verbal stimuli were recorded in a silent room by a female speaker, and the tonal stimuli (sine tones) were generated by a computer program. The audio files and experimental procedures were edited and presented using a laptop computer and earphones.

The experimental session had an average duration of 60 min. The participant received general information about the study, signed a consent form, answered questions about the inclusion criteria, and underwent audiological evaluation. If the inclusion criteria were met, the participant performed four memory tasks. Two main tasks were aimed at investigating tone and pseudoword recognition under interference conditions. Other two tasks were included for assessing the participants’ memory for tones and pseudowords separately: tonal sequence recognition and pseudoword sequence recall (since a pilot study showed that performance would be at ceiling levels with a pseudoword sequence recognition procedure). The order of presentation of the memory tasks was counterbalanced in a way that verbal and tonal tasks were presented alternately. At the beginning of each memory task, the participant read and heard the instructions and performed practice trials. Each trial started with a warning tone (4000 Hz for 50 ms), followed by a silent interval of 950 ms and the presentation of stimuli. The participants gave their answers verbally. The answers were tape-recorded, and the experimenter registered it on a response sheet immediately for later scoring. The experimenter was not blind to the correct answers.

#### Tonal sequence recognition

This task comprised the presentation of two sequences of seven sine tones separated by a silent interval of 3 s, and the task was to judge if the two sequences were the same or different. Each tone was presented for 300 ms, with an interstimulus interval of 50 ms. The tones were from the second and third octaves, and the frequency interval between consecutive tones in the sequences did not follow any specific musical rules (i.e., sequences with strong tonal structure were avoided, cf. Croonen, 1994), so that they would not be easily recognized by musicians. Each sequence consisted in an ascendant melodic line followed by a descendent one. In addition to two practice sequences, 14 sequences were used as stimuli in a total of 28 trials (see Appendix 1 for the stimuli). In half of the trials, the probed sequences were the same as the memorized sequences; in the other half, one single tone in the probed sequence changed. Changes could occur at any serial position with equal probability, and the different tone was always three semitones (above or below) from the original one. The number of correct responses (i.e., the number of correct “same” and “different” answers) was computed for statistical analysis.

#### Phonological sequence recall

In this task, a sequence of pseudowords was presented and then the participant was asked to repeat it back verbatim. Only disyllabic pseudowords were used and sequence length varied from two to six items, with two trials for each level of difficulty following a memory span procedure (see Appendix 2 for the list of stimuli). If the participant was able to repeat correctly at least one sequence of a given length, the two sequences of the following length were presented; otherwise, the testing stopped. The mean number of pseudowords recalled correctly was computed for statistical analysis.

#### Tonal and phonological recognition under irrelevant stimuli conditions

In these tasks, one stimulus was presented for memorization and the probe stimulus was presented after a retention interval of 5000 ms. There were three blocked experimental conditions during the retention interval (silence, irrelevant tones, and irrelevant speech). In the tonal recognition task, twelve pairs of sine tones each one lasting 200 ms were created for the experimental conditions. The tones for memorization were twelve notes: A3, A#, B3, C#3, C4, D#3, D3, E3, F#3, F3, G#3, and G3. In half of the trials, the probe was the same tone as the previously heard, and in the other half, it differed from it in one semitone (above or below) (see Appendix 3). In the pseudoword recognition task, twelve pairs of multisyllabic pseudowords (mean duration = 1500 ms) composed of simple consonant-vowel syllabic structure were elaborated for the experimental conditions, and in half of the trials, there was a slight phonetical difference between the pseudowords of a pair (see Appendix 4). In specific, the consonants of the central syllable differed in two types of phonetic features—place of articulation (e.g., bilabial, linguodental etc.) and voice (vibration or not of the vocal folds). For example, a central syllable “pa” became “da,” that is, the consonant [p] [bilabial, −voice] was substituted for [d] [linguodental, +voice]. In this way, the recognition task required the evaluation of the overall auditory-phonetical pattern of the pseudowords.

The presentation of irrelevant stimuli begun 300 ms after the offset of the main stimulus and ended 2000 ms before the probe stimulus (cf. Deutsch, 1970; Pechmann and Mohr, 1992). In the irrelevant tones condition, six tones of 200 ms separated by 300 ms were presented during the retention interval, and none of them were the same as the memorized tone within the same trial (see Appendix 3). In the irrelevant speech condition, four dissyllabic words were presented (see Appendix 4). For each condition, in half of the trials the probe was the same as the memorized stimulus, and in the other half, the probe was different. Memory recognition performance was assessed by the sensitivity index *d′* (Snodgrass & Corwin, 1988; Stanislaw & Todorov, 1999).

## Results

Table 1 shows the descriptive statistics for tonal sequence recognition and phonological sequence recall. For each task, we used the nonparametric Mann-Whitney *U* test to compare scores from two independent samples. Musicians were better than nonmusicians in the tonal task, *U* = 85, *Z* = 3.6, *p* < .001, *r* = .55, and there was no difference between the groups in the phonological task (*p* = .59, *r* = .09).

**Table 1:** Mean number of correct responses (M), with standard deviation (SD), median, and range (minimum-maximum) in tonal sequence recognition and pseudoword sequence recall tasks, for each group of participants

	Musicians (*n* = 21)			Nonmusicians (*n* = 22)		
Task	M (SD)	Median	Min–max	M (SD)	Median	Min–max
Tonal	19.9 (2.79)	20	14–24	16.5 (2.67)	16	9–21
Phonological	3.8 (0.71)	3.8	2.5–4.8	3.7 (0.78)	3.8	1.8–5.5

**Fig. 1:**
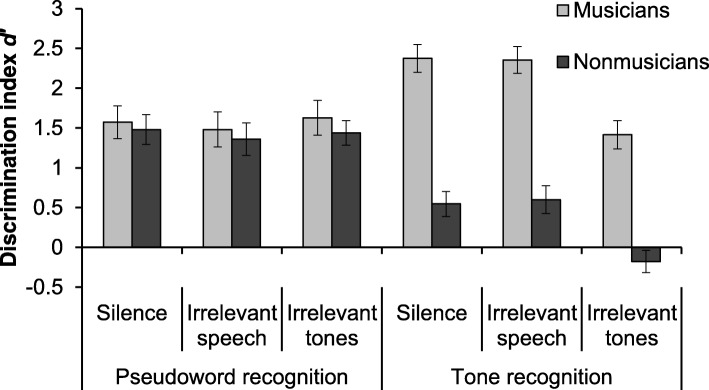
Mean proportion of correct responses in pseudoword and tone recognition by musicians and nonmusicians in silence or in presence of irrelevant speech or tones. Error bars denote standard errors of the mean

Performance in tonal and phonological recognition tasks under silence and interference conditions is summarized in Fig. 1. We carried out a mixed ANOVA with group (musicians vs. nonmusicians) as between-subjects factor, and task (phonological vs. tonal) and condition (silence vs. irrelevant speech vs. irrelevant tones) as within-subjects factors. A main effect of group was observed, *F*(1, 41) = 28.6, *p* < .001, η^2^_p_ = .41, given that musicians had a better overall performance (M = 1.80, SE = 0.124) than nonmusicians (M = 0.87, SE = 0.122). A main effect of task was also observed, *F*(1, 41) = 7.3, *p* = .010, η^2^_p_ = .15, resulting from the fact that pseudoword recognition (M = 1.49, SE = 0.115) was better than tone recognition (M = 1.19, SE = 0.092). A main effect of condition was also observed, *F*(2, 82) = 9.9, *p* < .001, η^2^_p_ = .19, and the post hoc test with Bonferroni’s correction showed that performance was significantly lower with irrelevant tones (M = 1.08, SE = 0.100) than both the silent (M = 1.49, SE = 0.102, *p* < .001) and the irrelevant speech conditions (M = 1.45, SE = 0.113, *p* = .002).

The factors group and task had a significant interaction, *F*(1, 41) = 48.5, *p* < .001, η^2^_p_ = .54, indicating that musicians and nonmusicians had different performances in the phonological and tonal recognition tasks. In fact, the post hoc test with Bonferroni’s correction revealed a significant discrepancy (*p* < .001) between musicians and nonmusicians in tonal recognition (M = 2.05, SE = 0.131, and M = 0.32, SE = 0.128, respectively), but not in pseudoword recognition (M = 1.56, SE = 0.165, and M = 1.43, SE = 0.161, respectively). A significant interaction was also observed for the factors task and condition, *F*(2, 82) = 19.2, *p* < .001, η^2^_p_ = .32, and the post hoc test with Bonferroni’s correction revealed no significant differences between the three conditions for the pseudoword recognition task, whereas the performance in tone recognition was significantly worse in the irrelevant tones condition (M = 0.62, SE = 0.112) than both the silent (M = 1.46, SE = 0.117) and the irrelevant speech (M = 1.48, SE = 0.122) conditions. Finally, the two-way interaction between group and condition (*F* < 1, *p* = .94) and the three-way interaction between group, task, and condition (*F* < 1, *p* = .61) were not significant, providing evidence that irrelevant tones impaired tone recognition for both groups of participants and that group differences concerned the overall performance of nonmusicians in tone recognition (cf. the significant interaction between group and task).

Given the poor performance of nonmusicians in the three conditions (silence, irrelevant words, and tones) of the tone recognition task (*d′* = 0.55, *d′* = 0.60, and *d′* = − 0.18, respectively), we carried out further analyses for discarding the possibility that the interference effect was not reliable for this group, that is, we verified whether performance in each condition was at chance. Since *d′* values close to zero indicate discrimination at chance, we used one-sample *t* tests to verify if each mean was different from zero. The mean performance in the silence and irrelevant words conditions were significantly different from zero, *t*(21) = 3.47, *p* = .002, and *t*(21) = 3.40, *p* = .003, respectively, indicating that performance was above chance in these conditions. In contrast, the mean performance in the irrelevant tones condition did not differ from zero, *t*(21) = − 1.27, *p* = .22, indicating performance at chance. Thus, we have consistent evidence that irrelevant tones disrupted the performance of nonmusicians in tone recognition.

## Discussion

The present study used the interference paradigm for investigating whether overlapping or dissociated WM processes underlie the temporary retention of phonological and tonal information and whether the results were similar for musicians and nonmusicians. To this purpose, pseudoword and tone recognition tasks were carried out in different retention interval conditions: silence, irrelevant words, or irrelevant tones. Considering the literature review, we hypothesized that pseudoword recognition would be impaired by irrelevant words and not by tones and that tone recognition would be impaired by irrelevant tones and not by words. Our results indicated that tone recognition was impaired by irrelevant tones for both groups of participants and that pseudoword recognition was not disrupted either by irrelevant words or tones. The groups did not differ significantly in pseudoword recognition, and musicians outperformed nonmusicians in tone recognition.

The present study also considered whether musicians and nonmusicians would differ in the other two tasks assessing the recognition of sequence of tones and the recall of a sequence of pseudowords. Musicians fared better than nonmusicians in the tonal sequence recognition task, and no group difference was observed for the pseudoword sequence recall task. Overall, our results showed that group differences concerned tonal memory and that our sample of musicians and nonmusicians did not differ in the tasks that involved verbal stimuli.

The results regarding group differences in the tone memory tasks are in line with evidence that musical training is associated with better performance in a range of musical and cognitive tasks (Schulze & Koelsch, 2012; Schulze et al., 2011; Williamson et al., 2010b) but are in contrast with evidence that it is associated with better performance in verbal WM tasks (Chan et al., 1998; Franklin et al., 2008; George & Coch, 2011; Hansen et al., 2013; Ho et al., 2003; Jakobson et al., 2008; Roden et al. 2014). It should be noted that musicians’ advantages in verbal memory tasks may be associated with better auditory temporal processing skills and more efficient extraction of semantic information during the encoding of information (Jakobson et al., 2008), as well as with more effective rehearsal (Franklin et al., 2008). In the current study, the use of dissyllabic and multisyllabic pseudowords may have posed higher demands on phonological processing in the absence of semantic (or lexical) information, and this could have challenged both groups of participants in a similar way. Since this study focused on investigating interference effects in pseudoword and tone recognition, we only included a tone sequence recognition and a pseudoword sequence recall tasks as additional measures of tonal and phonological WM, but we did not include other measures of WM in which musicians were shown to perform better than nonmusicians, such as the recall of words, digits, letters, and monosyllabic nonwords (Chan et al., 1998; Franklin et al., 2008; George & Coch, 2011; Hansen et al., 2013; Ho et al., 2003; Jakobson et al., 2008).

Regarding tonal and pseudoword recognition in conditions of silence or irrelevant words or tones, the results showed that performance in pseudoword recognition did not differ significantly between conditions, and tone recognition was disrupted only by irrelevant tones for both groups of participants, as revealed by the significant interaction between the factors task and condition in the absence of a significant three-way interaction with the factor group. Separate analyses confirmed the reliability of the tonal interference effect for nonmusicians, discarding the possibility that they fared equally poor (at chance) across the conditions.

Regarding pseudoword recognition, our study aimed to disrupt phonological codes within WM. We did not find the expected disruption of performance in the presence of interpolated verbal information. This result was unexpected because we devised a difficult recognition task that involved subtle phonetical/phonological differences, namely, slight changes in consonants in the central syllable of multisyllabic pseudowords. There was no evidence for displacement or overwriting of phonological codes by incoming irrelevant information, and this contrasts with evidence regarding disruption of word recognition by irrelevant words and tones (Atherton et al., 2018) and disruption due to a high overlap of phonetic features between memorized and interpolated items (Eagan & Chein, 2012). However, it is possible that the characteristics of the stimuli in our study prevented interference during the retention interval. In order to increase the task difficulty, we used multisyllabic pseudowords and the critical central syllable was followed by other syllables prior to the retention interval. Thus, it might be the case that some degree of interference occurred prior to the retention interval similarly across the experimental conditions. In contrast, Atherton et al. (2018) employed monosyllabic words as stimuli, with slight changes either in the first consonant or in the last one in the different probes. Although such word recognition task was easier than multisyllabic pseudoword recognition and had a semantic component (that might have protected memory from interference), it seemed adequate to investigate the interference effect.

It is worth noting that irrelevant sound/speech interference effects on phonological memory were mainly found using a serial recall procedure of visually presented linguistic items (e.g., Alley & Greene, 2008; Colle & Welsh, 1976; Iwanaga & Ito, 2002; Salame and Baddeley, 1982, Salamé and Baddeley, 1989) and that the lack of interference on pseudoword recognition is in line with the more usual interference-by-process account of the irrelevant sound/speech effects on phonological memory (Baddeley, 2007, 2012; Beaman & Jones, 1997, 1998; Jones & Macken, 1993; Jones & Tremblay, 2000; Jones et al., 1992). However, few studies aimed at exploring the possibility of phonological interference-by-content within WM (Atherton et al., 2018; Eagan & Chein, 2012), and further research is necessary to explore whether an auditory-verbal recognition task with a single stimulus is resistant or susceptible to disruption by interpolated speech during the retention interval. In particular, a promising way to investigate this issue would be to replicate the general design by Atherton et al. (2018) with monosyllabic nonwords (i.e., nonsense syllables) as to-be-memorized stimuli and interference stimuli, reducing the semantic component.

Regarding musicians, although they seemed to be able to take advantage from their musical knowledge, they were not able to cope with tonal interference, namely, to employ strategies to maintain their memory performance at the same level of the other conditions. In the study by Williamson et al. (2010a), their sample of musicians employed verbal or motor strategies to cope with tonal similarity (pitch-proximity). In our study, the use of verbal or motor strategies (such as naming the tone or creating a mental image of playing the tone) would have allowed musicians to recognize the tone even in the presence of distracting tones, but this was not the case. In our study, since the to-be-memorized tone lasted for 200 ms and there was a 300 ms interval to the onset of the interpolated sequence, the time was probably too short to allow the use of cognitive strategies for encoding the auditory stimulus. Thus, our results suggest that both musicians and nonmusicians relied on auditory WM to maintain tonal information active in the presence of irrelevant sounds, involving processes that are prone to tonal interference, but not to phonological interference (e.g., Jones et al., 1997).

Our study offers further evidence that memory for tones is susceptible to interference-by-content (i.e., by irrelevant tones) (see also Atherton et al., 2018; Deutsch, 1970; Jones et al. 1997; Pechmann & Mohr, 1992, Williamson et al., 2010a), which is consistent across participants differing in musical expertise in our sample. This consistency was in contrast with the results reported by Pechmann and Mohr (1992), who found that both verbal and visual material disrupted performance in tonal recognition for nonmusicians, and we did not find evidence of phonological interference on tone recognition for nonmusicians. According to Pechmann and Mohr (1992), the source of the interference patterns observed in their study was the distract of attention during the retention interval with interpolated visual or verbal material. In our study, the participants were instructed to focus on the memory task and to ignore any irrelevant sound during the retention interval, and both groups had difficulties in performing tone recognition with interpolated irrelevant tones, and this was not observed in the irrelevant speech condition. Thus, by using the interference paradigm, our study found evidence for interference-by-content for tonal WM that is independent of musical expertise.

As discussed in the introduction, disruption of tonal memory may derive from perceptual processes such as spectral similarity, timing, or spatial proximity (Jones et al. 1997; Semal et al., 1996), as well as from memory processes such as retroactive interference and feature overwriting (Mercer & McKeown, 2010a, 2010b). In contrast, disruption of phonological memory may mainly derive from interference-by-process (Baddeley, 2007, 2012; Beaman & Jones, 1997, 1998; Jones & Macken, 1993; Jones & Tremblay, 2000; Jones et al., 1992), but also may derive from interference-by-content (Atherton et al., 2018; Eagan & Chein, 2012), as should be further investigated in future research.

## Conclusions

In conclusion, the present study found evidence that the WM system involved in tonal recognition is associated with musical experience and is both susceptible to tonal interference and resistant to phonological interference, regardless of musical training. In contrast, pseudoword recognition was resistant to both tonal and phonological interference, arguably due to the syllables that followed the critical central syllable, and future research should investigate whether monosyllabic pseudowords are resistant or susceptible to auditory-verbal interference.

## Appendix 1

### Example of the stimuli of the tonal sequence recognition task

**Table 2:** The fourteen sequences of seven tones used in each one of the trials, with the type of test-sequence (the same or different as the memorized sequence) and the discrepant tone (above [↑] or below [↓] the respective original tone) in the “different” trials

Trial	Stimuli serial presentation							Test stimuli	
	1	2	3	4	5	6	7	Type	Change
1/15	F#2	**A2**	F3	C#3	B2	G#2	E2	Different	A2 → C3 ↑
2/16	C3	G#3	B3	A3	F3	D#3	G2	Same	**–**
3/17	B2	F3	A#3	**C4**	G#3	D3	G#2	Different	C4 → D#4 ↑
4/18	D3	A3	B3	F3	D#3	A2	G2	Same	**–**
5/19	**A#2**	D3	F3	C#4	B3	F3	E2	Different	A#2 → G2 ↓
6/20	E3	F3	A#3	G3	D#3	B2	D2	Same	**–**
7/21	D#2	A2	E3	C3	**A#2**	F#2	C2	Different	A#2 → G2 ↓
8/22	E2	A#2	**F3**	A#3	G3	C#3	F2	Different	F3 → G#3 ↑
9/23	A2	D#3	F3	B3	C3	A#2	G#2	Same	**–**
10/24	D#3	F3	B3	C4	F#3	A2	G2	Same	**–**
11/25	F2	B2	D3	C3	G#2	F#2	**E2**	Different	E2 → G2 ↑
12/26	G2	C#3	D3	C3	G#2	F2	D#2	Same	**–**
13/27	G#2	D3	A#3	E3	C3	A#2	F#2	Same	**–**
14/28	C#3	D3	G#3	B2	A#3	**E3**	A2	Different	E3 → G3 ↑

Note: Tones that were changed from the original sequence are marked in bold

## Appendix 2

### Example of the stimuli of the pseudoword sequence recall task

**Table 3:** The disyllabic pseudowords used in each one of the trials with their respective phonetic transcription

Trial	Number of stimuli	Stimuli serial presentation				
1	2	JETA ʒetɐ	NICA nikɐ			
2	2	ZARRI zari	GÔCA gokɐ			
3	3	DIMA d͡ʒimɐ	SICO siko	DEGUI dɛgi		
4	3	JOBA ʒɔbɐ	GUIME gimə	LARRO larʊ		
5	4	TILA t͡ʃilɐ	XUNA ʃunɐ	PADI pad͡ʒi	TEPU tɛpʊ	
6	4	PADU padʊ	JOBE ʒɔbə	DIRRA d͡ʒirɐ	GOBI gɔbɪ	
7	5	REFO rɛfʊ	PUME pumə	TABO tabo	GUILA gilɐ	JAFE ʒafə
8	5	LÉXA lɛʃɐ	DÊRRU derʊ	MÓPE mɔpə	GUSSI gusɪ	XONE ʃonə

## Appendix 3

### Example of stimuli of the tonal recognition task with irrelevant tones during the retention interval

**Table 4:** The twelve tones used as stimuli and the respective test stimuli, with the irrelevant tones that were presented during the retention interval. The test stimulus could be the same as the memorized one, or being a semitone above (↑) or below (↓) the memorized one in the “different” trials

Stimulus	Sequence of irrelevant stimuli						Test	Type
*F#3*	C3	G4	A3	B3	D4	A#3	F3 ↓	Different
E3	F#2	F4	A2	G3	B3	G#3	D#3 ↓	Different
F3	G#2	F#4	B2	C#3	A3	D4	E3 ↓	Different
G3	F4	F#4	D#4	C4	E3	B2	G#3 ↑	Different
A3	E4	G#4	C#4	F#3	D3	B2	A#3 ↑	Different
B3	G2	A#4	A2	C#3	D#3	F4	C4 ↑	Different
C4	G3	B4	F4	D#4	A3	G#3	C4	Same
C#3	G2	D4	A#2	D#3	F3	D4	C#3	Same
D3	C4	C#4	E4	A#3	B3	G3	D3	Same
A#3	F#2	A4	G#2	C3	D3	F3	A#3	Same
G#3	E4	A4	D4	D#3	C3	A2	G#3	Same
D#3	B2	E4	G#3	C4	E4	A#3	D#3	Same

## Appendix 4

### Example of stimuli of the pseudoword recognition task with irrelevant words during the retention interval

**Table 5:** The twelve pseudowords used as stimuli and the respective test stimuli, with the irrelevant words that were presented during the retention interval. Phonetic transcriptions are provided below the stimuli. The test stimulus could be the same as the memorized one, or the consonant of the central syllable was changed in the “different” trials

Stimulus	Sequence of irrelevant stimuli				Test	Type
BEMACU*T*EZAPIRRE bemakutezapirə	mola mɔlɐ	calça kaʊsɐ	milho miʎʊ	alho aʎʊ	BEMACU**B**EZAPIRRE bemakubezapirə	Different
CAZEMO*GU*EDOLINE kazemɔgedolinə	pato patʊ	casa kazɐ	bico bikʊ	porta portɐ	CAZEMO**T**EDOLINE kazemɔtedolinə	Different
MAFIZU*QU*EBANICA mafizukebɑ̃nikɐ	sala salɐ	anel anɛʊ	fita fitɐ	moça mosɐ	MAFIZU**D**EBANICA mafizudebɑ̃nikɐ	Different
SAMOTE*B*UCADILA sɑ̃mɔtebukad͡ʒilɐ	perna pɛɾnɐ	lama lɑ̃mɐ	lenço lensʊ	pote pɔt͡ʃɪ	SAMOTE**T**UCADILA sɑ̃mɔtetukad͡ʒilɐ	Different
SICAJE*D*AZUTAGA sikaʒedazutagɐ	olho oʎʊ	carne karnə	cama kɑ̃mɐ	vaso vasʊ	SICAJE**P**AZUTAGA sikaʒepazutagɐ	Different
XIDESSA*P*EGAVUBA ʃidesapegavubɐ	perna pɛrnɐ	mesa mesɐ	chave ʃavɪ	pinça pinsɐ	XIDESSA**D**EGAVUBA ʃidesadegavubɐ	Different
LAXIFOTARRISSABA laʃifotarisabɐ	copo kɔpʊ	lapis lapis	galo galʊ	rato ħatʊ	LAXIFOTARRISSABA laʃifotarisabɐ	Same
MEGORUGASSALOVA meɢoɾugasalovɐ	cola kɔlɐ	moto mɔtʊ	disco diskʊ	ponta pontɐ	MEGORUGASSALOVA meɢoɾugasalovɐ	Same
PATELACORRUBEZI patelakorubɛzɪ	boca bokɐ	lata latɐ	vara varɐ	peixe peɪʃɪ	PATELACORRUBEZI patelakorubɛzɪ	Same
TORROVEPOLASSEPA torovɛpolasepɐ	mula mulɐ	loja lɔʒɐ	foto fɔtʊ	colar kolaɾ	TORROVEPOLASSEPA torovɛpolasepɐ	Same
TOVEGUBASSARIFA tovegubasaɾifɐ	mala malɐ	prato pɾatʊ	chapa ʃapɐ	maçã masɑ̃	TOVEGUBASSARIFA tovegubasaɾifɐ	Same
ZICANEPEFOTAVI zikanepefotavɪ	chapéu ʃapɛʊ	sapo sapʊ	capa kapɐ	sela sɛlɐ	ZICANEPEFOTAVI zikanepefotavɪ	Same

Note. Consonants that changed from the original stimulus (in italics) are indicated in bold in the test stimulus

## Abbreviation

WM: Working memory

## References

[CR1] Alley TR, Greene ME (2008). The relative and perceived impact of irrelevant speech, vocal music and non-vocal music on working memory. Current Psychology.

[CR2] American Speech-Language-Hearing Association (1978). Guidelines for manual pure-tone threshold audiometry.

[CR3] Atherton RP, Chrobak QM, Rauscher FH, Karst AT, Hanson MD, Steinert SW, Bowe KL (2018). Shared processing of language and music. Experimental Psychology.

[CR4] Baddeley AD (2007). Working memory, thought, and action.

[CR5] Baddeley AD (2012). Working memory: theories, models, and controversies. Annual Review of Psychology.

[CR6] Beaman CP, Jones DM (1997). Role of serial order in the irrelevant speech effect: tests of the changing-state hypothesis. Journal of Experimental Psychology: Learning, Memory, and Cognition.

[CR7] Beaman, C. P., & Jones, D. M. (1998). Irrelevant sound disrupts order information in free recall as in serial recall.* Quarterly Journal of Experimental Psychology Section A*, *51*(3), 615–636. 10.1080/713755774. 10.1080/713755774 9745380

[CR8] Benassi-Werke, M. E., Queiroz, M., Araujo, R. S., Bueno, O. F., & Oliveira, M. G. M. (2012). Musicians’ working memory for tones, words, and pseudowords. *Quarterly Journal of Experimental Psychology*, *65*(6), 1161–1171. 10.1080/17470218.2011.644799. 10.1080/17470218.2011.644799 22352405

[CR9] Berz WL (1995). Working memory in music: a theoretical model. Music Perception.

[CR10] Chan AS, Ho YC, Cheung MC (1998). Music training improves verbal memory. Nature.

[CR11] Chein, J. M., & Fiez, J. A. (2010). Evaluating models of working memory through the effects of concurrent irrelevant information. *Journal of Experimental Psychology: General*, *139*(1), 117–137. 10.1037/a0018200. 10.1037/a0018200 PMC2849630 20121315

[CR12] Colle HA, Welsh A (1976). Acoustic masking in primary memory. Journal of Verbal Learning and Verbal Behavior.

[CR13] Croonen WLM (1994). Effects of length, tonal structure, and contour in the recognition of tone series. Perception & Psychophysics.

[CR14] Davis H, Silverman RS (1978). Hearing and deafness.

[CR15] Deutsch D (1970). Tones and numbers: specificity of interference in immediate memory. Science.

[CR16] Eagan DE, Chein JM (2012). Overlap of phonetic features as a determinant of the between-stream phonological similarity effect. Journal of Experimental Psychology: Learning, Memory, and Cognition.

[CR17] Franklin MS, Moore KS, Yip CY, Jonides J, Rattray K, Moher J (2008). The effects of musical training on verbal memory. Psychology of Music.

[CR18] George EM, Coch D (2011). Music training and working memory: an ERP study. Neuropsychologia.

[CR19] Hansen M, Wallentin M, Vuust P (2013). Working memory and musical competence of musicians and non-musicians. Psychology of Music.

[CR20] Ho YC, Cheung MC, Chan AS (2003). Music training improves verbal but not visual memory: cross-sectional and longitudinal explorations in children. Neuropsychology.

[CR21] Iwanaga M, Ito T (2002). Disturbance effect of music on processing of verbal and spatial memories. Perceptual and Motor Skills.

[CR22] Jakobson LS, Lewycky ST, Kilgour AR, Stoesz BM (2008). Memory for verbal and visual material in highly trained musicians. Music Perception.

[CR23] Jones, D. M., & Macken, W. J. (1993). Irrelevant tones produce an irrelevant speech effect: implications for phonological coding in working memory. *Journal of Experimental Psychology: Learning, Memory, and Cognition*, *19*(2), 369–381. 10.1037/0278-7393.19.2.369.

[CR24] Jones, D. M., & Macken, W. J. (1995). Phonological similarity in the irrelevant speech effect: within-or between-stream similarity? *Journal of Experimental Psychology: Learning, Memory, and Cognition*, *21*(1), 103–115. 10.1037/0278-7393.21.1.103.

[CR25] Jones, D. M., Macken, W. J., & Harries, C. (1997). Disruption of short term recognition memory for tones: streaming or interference? *Quarterly Journal of Experimental Psychology Section A*, *50*(2), 337–357. 10.1080/713755707. 10.1080/713755707 9225626

[CR26] Jones, D. M., Madden, C., & Miles, C. (1992). Privileged access by irrelevant speech to short-term memory: the role of changing state. *Quarterly Journal of Experimental Psychology Section A*, *44*(4), 645–669. 10.1080/14640749208401304. 10.1080/14640749208401304 1615168

[CR27] Jones, D. M., & Tremblay, S. (2000). Interference in memory by process or content? A reply to Neath (2000). *Psychonomic Bulletin & Review*, *7*(3), 550–558. 10.3758/BF03214370. 10.3758/bf03214370 11082864

[CR28] Koelsch S, Schulze K, Sammler D, Fritz T, Müller K, Gruber O (2009). Functional architecture of verbal and tonal working memory: an FMRI study. Human Brain Mapping.

[CR29] Larsen JD, Baddeley A, Andrade J (2000). Phonological similarity and the irrelevant speech effect: implications for models of short-term verbal memory. Memory.

[CR30] LeCompte, D. C., & Shaibe, D. M. (1997). On the irrelevance of phonological similarity to the irrelevant speech effect. *Quarterly Journal of Experimental Psychology Section A*, *50*(1), 100–118. 10.1080/713755679. 10.1080/713755679 9080790

[CR31] Mercer, T., & McKeown, D. (2010a). Interference in short-term auditory memory. *Quarterly Journal of Experimental Psychology*, *63*(7), 1256–1265. 10.1080/17470211003802467. 10.1080/17470211003802467 20446187

[CR32] Mercer, T., & McKeown, D. (2010b). Updating and feature overwriting in short-term memory for timbre. *Attention, Perception, & Psychophysics*, *72*(8), 2289–2303. 10.3758/APP.72.8.2289. 10.3758/bf03196702 21097870

[CR33] Page, M. P. A., & Norris, D. G. (2003). The irrelevant sound effect: what needs modelling, and a tentative model. *Quarterly Journal of Experimental Psychology Section A*, *56*(8), 1289–1300. 10.1080/02724980343000233. 10.1080/02724980343000233 14578085

[CR34] Pechmann T, Mohr G (1992). Interference in memory for tonal pitch: Implications for a working-memory model. Memory & Cognition.

[CR35] Pereira LD, Schochat E (1997). Manual de avaliação do processamento auditivo central [Administration manual of central auditory processing].

[CR36] Roden I, Grube D, Bongard S, Kreutz G (2014). Does music training enhance working memory performance? Findings from a quasi-experimental longitudinal study. Psychology of Music.

[CR37] Salame, P., & Baddeley, A. (1982). Disruption of short-term memory by unattended speech: Implications for the structure of working memory. *Journal of Verbal Learning and Verbal Behavior*, *21*(2), 150–164. 10.1016/S0022-5371(82)90521-7.

[CR38] Salamé, P., & Baddeley, A. (1989). Effects of background music on phonological short-term memory. *Quarterly Journal of Experimental Psychology Section A*, *41*(1), 107–122. 10.1080/14640748908402355.

[CR39] Santos TMM, Russo ICP (1986). *A prática da audiologia clínica* [the practice of clinical audiology].

[CR40] Schendel ZA, Palmer C (2007). Suppression effects on musical and verbal memory. Memory & Cognition.

[CR41] Schulze, K., & Koelsch, S. (2012). Working memory for speech and music. *Annals of the New York Academy of Sciences*, *1252*(1), 229–236. 10.1111/j.1749-6632.2012.06447.x. 10.1111/j.1749-6632.2012.06447.x 22524364

[CR42] Schulze K, Zysset S, Mueller K, Friederici AD, Koelsch S (2011). Neuroarchitecture of verbal and tonal working memory in nonmusicians and musicians. Human Brain Mapping.

[CR43] Semal, C., Demany, L., Ueda, K., & Hallé, P. A. (1996). Speech versus nonspeech in pitch memory. *Journal of the Acoustical Society of America*, *100*(2), 1132–1140. 10.1121/1.416298. 10.1121/1.416298 8759966

[CR44] Snodgrass, J. G., & Corwin, J. (1988). Pragmatics of measuring recognition memory: applications to dementia and amnesia. J*ournal of Experimental Psychology: General, 117*(1), 34–50. 10.1037/0096-3445.117.1.34. 10.1037//0096-3445.117.1.34 2966230

[CR45] Stanislaw, H., & Todorov, N. (1999). Calculation of signal detection theory measures. *Behavior Research Methods, Instruments, & Computers, 31*(1), 137–149. 10.3758/BF03207704. 10.3758/bf03207704 10495845

[CR46] Tremblay, S., Parmentier, F. B., Hodgetts, H. M., Hughes, R. W., & Jones, D. M. (2012). Disruption of verbal-spatial serial memory by extraneous air-traffic speech. *Journal of Applied Research in Memory and Cognition*, *1*(2), 73–79. 10.1016/j.jarmac.2012.04.004.

[CR47] Williamson VJ, Baddeley AD, Hitch GJ (2010). Musicians’ and nonmusicians’ short-term memory for verbal and musical sequences: comparing phonological similarity and pitch proximity. Memory & Cognition.

[CR48] Williamson, V. J., Mitchell, T., Hitch, G. J., & Baddeley, A. D. (2010b). Musicians’ memory for verbal and tonal materials under conditions of irrelevant sound. *Psychology of Music*, *38*(3), 331–350. 10.1177/0305735609351918.

[CR49] Zhang, J. D., Susino, M., Mcpherson, G. E., & Schubert, E. in press The definition of a musician in music psychology: a literature review and the six-year rule. *Psychology of Music*. 10.1177/0305735618804038.

